# Sepsis and acute kidney injury-related mortality in the U.S.: National trends and disparities (1999–2023)

**DOI:** 10.1097/MD.0000000000049495

**Published:** 2026-06-26

**Authors:** Rahul Balach, Shahtaj Tariq, Muhammad Taha Nizami, Muhammad Ibrahim, Hasibullah Aminpoor, Muhammad Ahsan, Shafaq Jawed, Adnan Safi, Saad Ahmed Waqas

**Affiliations:** a Department of Medicine, Ziauddin University, Karachi, Sindh, Pakistan; b Department of Medicine, Dow University of Health Sciences, Karachi, Sindh, Pakistan; c Department of Internal Medicine, Rabia Balkhi National Complex Hospital, Kabul, Afghanistan; d Faculty of Medicine, Kabul University of Medical Sciences “Abu Ali Ibn Sina”, Kabul, Afghanistan; e Department of Medicine, HCA Houston Healthcare Kingwood, Houston, TX; f Department of Nephrology, SUNY Downstate Health Sciences University, Brooklyn, NY.

**Keywords:** acute kidney injury, CDC WONDER, Healthcare Disparities, Mortality Trends, Public Health Equity, Sepsis

## Abstract

This study aimed to assess national mortality trends involving concurrent sepsis and acute kidney injury (AKI) documented on death certificates in the United States, with a focus on identifying temporal changes and disparities across demographic and geographic subgroups. Using Centers for Disease Control and Prevention’s Wide-ranging Online Data for Epidemiologic Research’s Multiple Cause-of-Death dataset (1999–2023), mortality trends in adults aged ≥ 25 years with concurrent sepsis and AKI documented on death certificates, identified via International Classification of Diseases, 10th Revision codes. Age-adjusted mortality rates were computed and stratified by sex, race/ethnicity, census region, urbanization, and age. Joinpoint regression analysis identified significant trend changes over time. 412,494 deaths involving concurrent sepsis and AKI were identified. Age-adjusted mortality rates rose from 3.51 to 13.11 per 100,000, with the sharpest increase occurring post-2018 (average annual percentage change: 6.43%, 95% confidence interval: 4.07–8.86). Mortality disproportionately affected older adults (≥ 65 years), males, Black and American Indian/Alaska Native populations, and rural residents. The South had the highest regional mortality, while the West experienced the steepest growth. Mortality rates increased substantially during the pandemic period. Geographic, racial, and socioeconomic disparities persisted throughout the study period. Mortality involving concurrent sepsis and AKI documented on death certificates increased over the study period, with accelerating trends and inequitable distribution among vulnerable groups. Pandemic-period increases may have contributed to the observed rise in mortality. These findings highlight the importance of improving early recognition, equitable healthcare access, and prevention strategies among high-risk populations.

**Key Points**

- From 1999 to 2023, 412,494 deaths involving concurrent sepsis and acute kidney injury (AKI) were identified. Age-adjusted mortality rates (AAMRs) rose from 3.51 to 13.11 per 100,000, with a marked post-2018 spike peaking in 2022.
- Males, older adults (≥ 65 years), and racial minorities (particularly Black and American Indian/Alaska Native populations) experienced significantly higher sepsis and AKI mortality rates.
- The South had the highest regional AAMR, while the West showed the steepest growth. State-level disparities were substantial, with Texas reporting the highest and Vermont the lowest average AAMRs.
- Nonmetropolitan (rural) areas consistently had higher mortality rates than metropolitan areas, especially after 2018, potentially reflecting disparities in access to critical care and nephrology services.
- Mortality involving concurrent sepsis and AKI increased substantially during the pandemic period, delaying care and disproportionately affecting racial minorities and rural populations.
- The findings support consideration of equity-focused strategies (including early warning systems, chronic disease management, and improved access to specialty care) to address the growing and unequal burden of sepsis and AKI-related mortality.

## 1. Introduction

Sepsis is a clinical syndrome that affects approximately 19 million people globally each year, including about 1.7 million individuals in the United States (U.S.).^[1,2]^ It is characterized by an innate immune response to infection, resulting in hypercoagulability and increased endothelial permeability due to a pro-inflammatory cascade.^[3]^ These changes lead to cytokine-mediated vasodilation of the renal vasculature, creating a mismatch between blood flow and demand. Additionally, compromised perfusion, mitochondrial dysfunction, and oxidative stress contribute to glomerular injury.^[4]^

Among the organs affected, acute kidney injury (AKI) develops in 25 to 75% of critically ill septic patients, with an in-hospitalmortality rate around 25%.^[5]^ Patients with both sepsis and AKI (SA-AKI) generally have a poorer prognosis and a higher sequential organ failure assessment score compared to patients with other organ involvement.^[6]^ Additionally, patients with recoverable AKI have a reported mortality rate of 28%, compared to 40% in the population with nonrecovery of renal function after sepsis.^[7]^ The presence of both conditions increases the likelihood of in-hospital death by 1.48 times.^[8]^ In addition to the attributed morbidity and mortality, SA-AKI is associated with higher healthcare costs due to increased intensive care unit utilization.^[9]^

Current understanding of demographic and comorbidity predictors for SA-AKI-related mortality is limited by varying definitions. Available data suggest higher mortality among older adults, Black individuals, and those with comorbidities such as diabetes, chronic kidney disease, and liver cirrhosis.^[1]^

Despite the growing burden of SA-AKI-related mortality, the current literature remains inadequately explored regarding its contemporary trends and demographics. Therefore, this study aims to analyze mortality rates and racial trends within the U.S. population affected by SA-AKI from 1999 to 2023 using data from the Centers for Disease Control and Prevention’s (CDC) Wide-ranging Online Data for Epidemiologic Research (CDC WONDER). These findings will guide equitable resource allocation and help formulate targeted strategies to reduce risks and improve health outcomes.

## 2. Methods

This study used de-identified, publicly accessible data from a government database and was therefore exempt from institutional review board approval. Informed consent was not required. The study follows the Strengthening the Reporting of Observational Studies in Epidemiology guidelines.

### 2.1. Study setting and population

This longitudinal study examined national mortality trends associated with SA-AKI using the CDC WONDER dataset. Specifically, we utilized the Multiple Cause of Death public-use dataset, which compiles death certificate data from all 50 U.S. states and the District of Columbia for the period 1999 to 2023. This dataset includes cause of death, demographic characteristics, and geographic classifications such as urbanization level and census region, and has been validated in multiple epidemiologic studies.

Our analysis focused on adults aged ≥ 25 years in whom sepsis was listed as either the underlying or a contributing cause of death within the multiple cause-of-death fields of the death certificate. Sepsis was identified using International Classification of Diseases, Tenth Revision (ICD-10) codes A40–A41, while concurrent AKI was identified using ICD-10 code N17 recorded anywhere on the same death certificate. Thus, cases represented deaths involving both sepsis and AKI, rather than confirmed causally linked SA-AKI events.^[10]^ Accordingly, the identified cohort represents deaths involving concurrent sepsis and AKI documented within the multiple cause-of-death fields rather than clinically adjudicated primary sepsis-induced AKI cases. Because CDC WONDER lacks laboratory and longitudinal clinical data, AKI identification relied exclusively on physician-reported ICD-10 coding recorded on death certificates. These ICD codes have been widely used in prior studies evaluating SA-AKI-related mortality using the CDC WONDER database.^[11]^ The study population was restricted to adults aged ≥ 25 years because CDC WONDER age-adjusted mortality rates (AAMRs) are based on predefined 10-year age groupings, with the first standard adult category beginning at 25 to 34 years. Inclusion of individuals aged 18 to 24 years would have required incorporation of the broader 15- to 24-year age category, thereby including pediatric and adolescent populations outside the intended adult study cohort.

### 2.2. Data abstraction

We extracted data on key demographic and geographic variables, including age, sex, race/ethnicity, census region, urban-rural status, and place of death. Age groups were categorized as young adults (25–44 years), middle-aged adults (45–64 years), and older adults (≥ 65 years). Sex was classified as male or female. Race and ethnicity categories included non-Hispanic (NH) White, NH Black or African American, NH American Indian or Alaska Native, and Hispanic or Latino. Place of death was classified as medical facilities, the decedent’s home, hospice facilities, or nursing homes/long-term care. Urbanization level was determined using the 2013 National Center for Health Statistics Urban-Rural Classification Scheme,^[12]^ and geographic regions were defined according to U.S. Census Bureau classifications: Northeast, Midwest, South, and West.^[13]^ For underlying cause of death, we used the “rankable cause of death” feature in CDC WONDER, which identifies mortality counts and rates for the 15 leading causes of death among individuals with these conditions.

### 2.3. Statistical analysis

We calculated AAMRs per 100,000 individuals, standardized to the 2000 U.S. standard population to account for demographic shifts over time.

Temporal trends were analyzed using the Joinpoint Regression Program (version 4.9.1.0; National Cancer Institute),^[14]^ which applies segmented log-linear regression models to annual AAMRs to detect statistically significant changes over time. For each segment, the annual percentage change (APC) and corresponding 95% confidence intervals (CIs) were calculated. The average annual percentage change (AAPC) summarized overall trends across the study period. Statistical significance was defined as a 2-tailed *P* value < .05.

## 3. Results

From 1999 to 2023, a total of 412,494 SA-AKI-related deaths were identified (Table 1). Males accounted for 51.46% of deaths. Most deaths occurred among White individuals (299,397), followed by Black or African American (58,242), Hispanic or Latino (37,457), and American Indian or Alaska Native individuals (3647). Older adults (≥ 65 years) represented the majority of deaths (74.54%), while most deaths occurred in medical facilities (93.03%) (Tables S1 and S2, Supplemental Digital Content 1 and 2).

**Table 1:** SA-AKI-associated mortality among U.S. from 1999 to 2023, stratified by sex, race, census region and location of death.

	Deaths	Population	AAMR 1999 (95% CI)	AAMR 2023 (95% CI)	AAPC (95% CI)
Overall	412,494	5,163,131,262	3.51 (3.43–3.6)	13.11 (12.97–13.25)	6.43 (4.07–8.86)
Sex					
Male	212,254	2,491,041,251	4.62 (4.46–4.78)	15.22 (15–15.44)	6.84 (5.05–8.66)
Female	200,240	2,672,090,011	2.76 (2.66–2.86)	11.45 (11.28–11.63)	5.48 (4.70–6.26)
Race					
American Indian or Alaska Native	3647	37,910,158	4.89 (3.34–6.9)	19.96 (17.77–22.15)	7.14 (3.16–11.26)
Black or African American	58,242	602,204,655	6.97 (6.54–7.4)	18.03 (17.51–18.54)	4.28 (3.20–5.38)
White	299,397	3,528,912,956	3.1 (3.01–3.19)	12.96 (12.79–13.12)	6.98 (4.93–9.06)
Hispanic or Latino	37,457	702,028,728	3.77 (3.35–4.2)	12.01 (11.6–12.42)	4.18 (2.16–6.24)
Census region					
Northeast	62,542	948,225,258	3.72 (3.53–3.92)	9.73 (9.45–10)	4.58 (2.78–6.40)
Midwest	82,208	1,110,999,093	3.2 (3.03–3.37)	12.03 (11.74–12.32)	6.54 (4.88–8.22)
South	177,071	1,916,380,109	3.84 (3.69–3.99)	15.13 (14.89–15.37)	6.15 (5.19–7.12)
West	90,673	1,187,526,802	3.03 (2.85–3.22)	13.5 (13.21–13.8)	7.28 (4.69–9.94)
Age					
Young Adults	14,917	2,119,405,590	0.36 (0.32–0.41)	1.62 (1.53–1.7)	6.95 (5.04–8.90)
Middle-Aged Adults	90,106	1,942,357,841	1.75 (1.64–1.86)	8.46 (8.27–8.66)	6.61 (4.41–8.86)
Older Adults	307,471	1,101,367,831	14.04 (13.65–14.44)	48.39 (47.81–48.98)	6.11 (4.44–7.80)
Place of Death					
Medical Facility	380,436	-	-	-	-
Decedent’s home	6511	-	-	-	-
Hospice facility	10,145	-	-	-	-
Nursing home/long-term care	11,827	-	-	-	-

### 3.1. Annual trends for Sepsis and AKI-related AAMR

**Figure 1.**
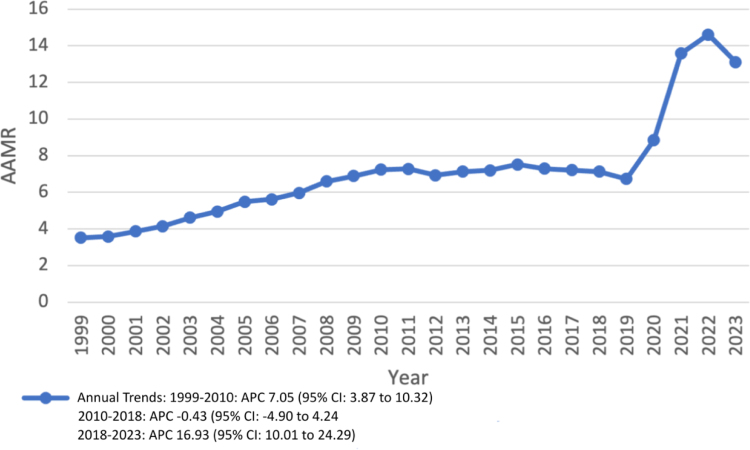
Annual SA-AKI-related AAMRs per 100,000 in the U.S., 1999 to 2023. AAMR = age-adjusted mortality rate, APC = annual percentage change, CI = confidence interval, SA-AKI = sepsis and acute kidney injury, U.S. = United States.

Overall, the AAMR showed a mostly stable upward trend for most of the period, with a sharp increase near the end when mortality rates rose significantly. It increased from 3.51 in 1999 to 13.11 in 2023 (AAPC: 6.43% [95% CI: 4.07–8.86%]). There was a steady rise from 1999 to 2010 (APC: 7.05% [95% CI: 3.87–10.32%]), followed by a plateau phase from 2010 to 2018 (APC: −0.43% [95% CI: −4.90–4.24%]) and then a sharp increase from 2018 to 2023 (APC: 16.93% [95% CI: 10.01–24.29%]), where the AAMR peaked in 2022 at 14.6 (Fig. 1, Table S3, Supplemental Digital Content 3, Table S8, Supplemental Digital Content 8). To further evaluate the potential influence of the coronavirus disease 2019 (COVID-19) pandemic on temporal mortality trends, a sensitivity analysis excluding pandemic-period years (2020–2023) was performed. In this analysis, SA-AKI-related AAMRs increased significantly from 1999 to 2010 (APC: 7.09%; 95% CI: 6.37–7.81%), followed by a stable to slightly declining trend between 2010 and 2019 (APC: −0.52%; 95% CI: −1.21–0.17%) with an overall AAPC: 3.59%; 95% CI: 3.13–4.05%. These findings suggest that the marked acceleration observed in the primary analysis was predominantly driven by pandemic-era mortality increases rather than a substantial rise during the immediate pre-pandemic period (Fig, S1, Supplemental Digital Content 10).

### 3.2. Sepsis and AKI-related AAMR stratified by sex

Among males, the AAMR increased from 4.62 in 1999 to 8.59 in 2010 (APC: 6.40% [95% CI: 5.83–6.97%]), followed by a decline from 2010 to 2018 (APC: −1.49% [95% CI: −2.27–− 0.71%]). Rates then rose sharply, reaching 16.66 in 2021 (APC: 26.77% [95% CI: 20.32–33.57%]), before stabilizing between 2021 and 2023 (APC: 0.30% [95% CI: −3.47–4.21%]), peaking at 17.42 in 2022 and declining to 15.22 in 2023.

**Figure 2.**
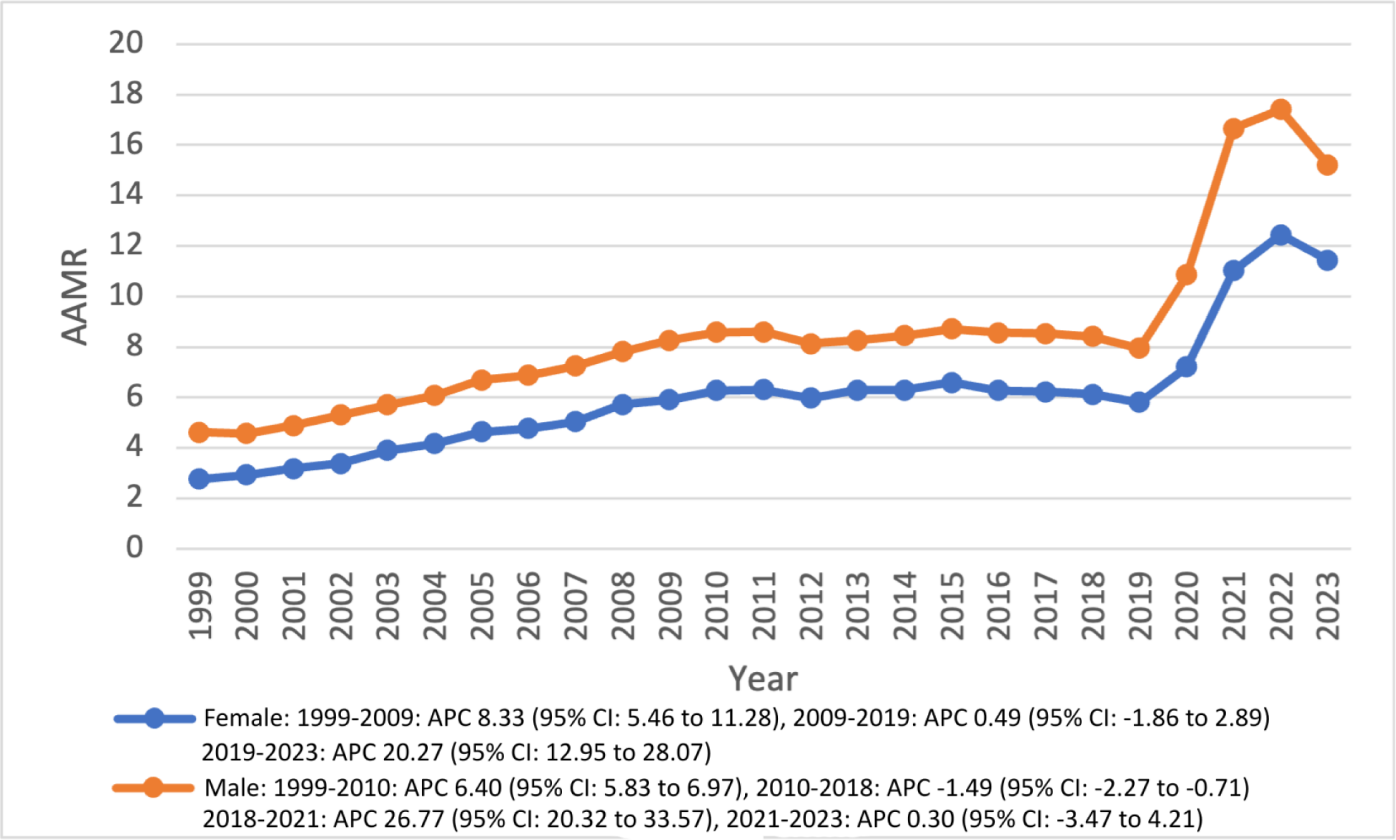
SA-AKI-related AAMRs per 100,000 stratified by gender in the U.S., between 1999 and 2023. AAMR = age-adjusted mortality rate, APC = annual percentage change, CI = confidence interval, SA-AKI = sepsis and acute kidney injury, U.S. = United States.

Females showed a similar but overall lower trend. The AAMR increased from 2.76 in 1999 to 5.91 in 2009 (APC: 8.33% [95% CI: 5.46–11.28%]), remained relatively stable from 2009 to 2019 (APC: 0.49% [95% CI: −1.86–2.89%]), and then increased markedly from 2019 to 2023, peaking at 12.44 in 2022 (APC: 20.27% [95% CI: 12.95–28.07%]) (Fig. 2, Table S3, Supplemental Digital Content 3, Table S8, Supplemental Digital Content 8; Fig. S1, Supplemental Digital Content 10).

### 3.3. Sepsis and AKI-related AAMR stratified by race

Among NH American Indian or Alaska Native individuals, the mortality trend fluctuated periodically, increasing steadily from 4.89 in 1999 to 12.1 in 2014 (APC: 8.08% [95% CI: 6.09–10.12%]), then a nonsignificant decrease to 10.24 in 2018 (APC: −7.40% [95% CI: −18.87–5.69%]). It then sharply increased to 24.54 in 2021 (APC: 36.71% [95% CI: 8.15–72.80%]) before slightly declining to 19.96 in 2023 (APC: −6.87% [95% CI: −22.42–11.79%]).

Among NH Black or African American individuals, the trend showed an overall increase from 1999 to 2023, with AAMR rising from 6.97 to 18.03 (AAPC: 4.28% [95% CI: 3.20–5.38%]). There was a slight decline between 2008 and 2018, when AAMR fell from 10.99 to 9.44 (APC: −2.56% [95% CI: −3.32%–−1.80%]), but reached a peak of 20.7 in 2022 (APC: 28.59% [95% CI: 19.39–38.51%]).

Among NH White individuals, the trend showed a steady increase in rates year over year. Rates increased from 3.1 in 1999 to 6.47 in 2009 (APC: 8.23% [95% CI: 4.80–11.76%]), followed by a very gradual rise over the next decade before returning to 6.47 in 2019 (APC: 0.84% [95% CI: −1.74–3.48%]). Afterward, rates accelerated to a maximum of 12.96 in 2023 (APC: 20.46% [95% CI: 12.32–29.20%]).

**Figure 3.**
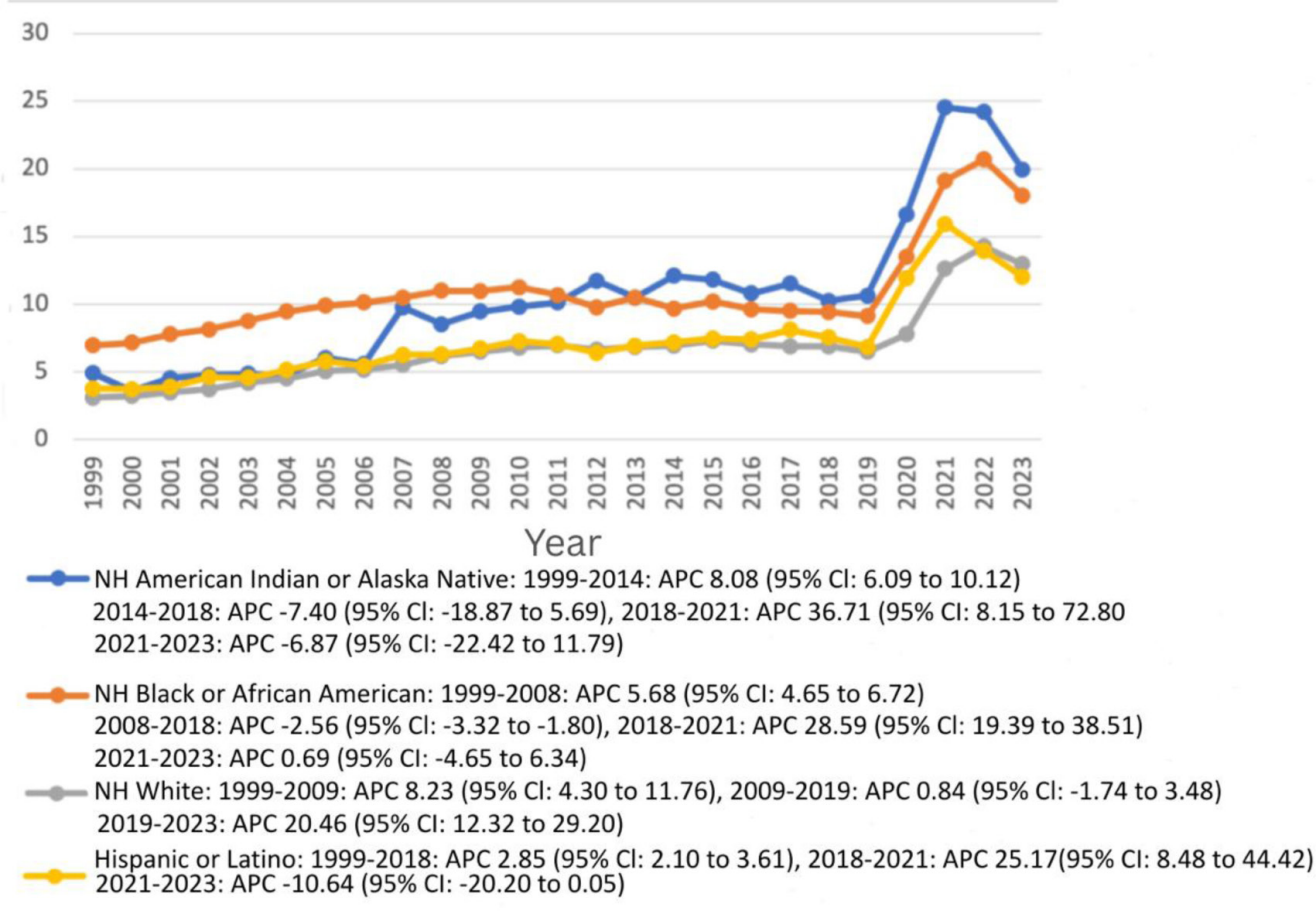
SA-AKI-related AAMRs per 100,000 stratified by race in the U.S., between 1999 to 2023. AAMR = age-adjusted mortality rate, APC = annual percentage change, CI = confidence interval, NH = non-Hispanic, SA-AKI = sepsis and acute kidney injury, U.S. = United States.

Finally, among Hispanic or Latino individuals, the trends showed a consistent increase from 3.77 in 1999 to 7.53 in 2018 (APC: 2.85% [95% CI: 2.10–3.61%]). This was followed by a sharp rise to 15.92 in 2021 (APC: 25.17% [95% CI: 8.48–44.42%]) and a decline to 12.01 in 2023 (APC: −10.64% [95% CI: −20.20–0.05%]). (Fig. 3, Table S4, Supplemental Digital Content 4, Table S8, Supplemental Digital Content 8) (Fig. S1, Supplemental Digital Content 10).

### 3.4. Sepsis and AKI-related AAMR stratified by census region

**Figure 4.**
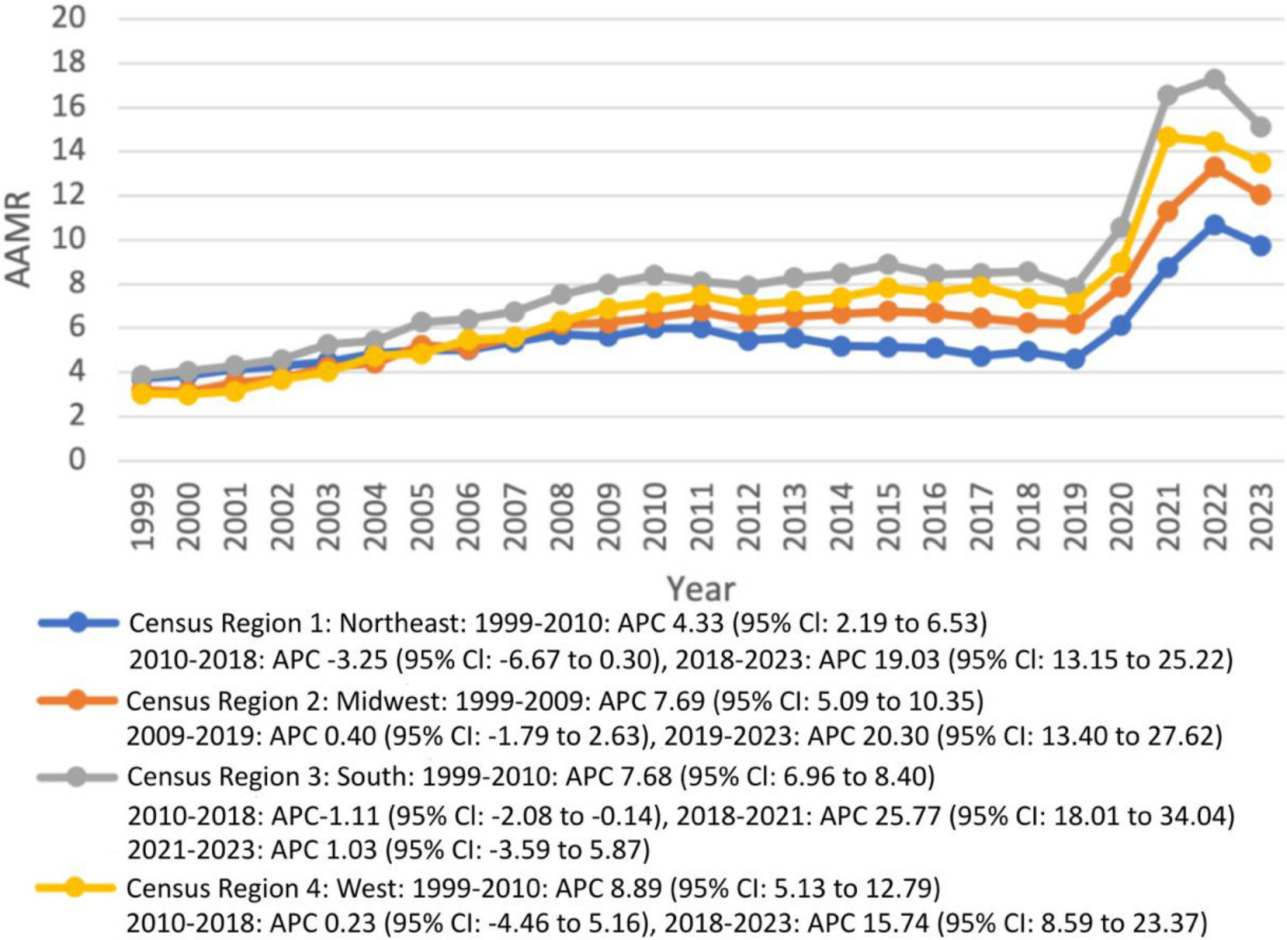
SA-AKI-related AAMRs per 100,000 stratified by census region in the U.S., between 1999 and 2023. AAMR = age-adjusted mortality rate, APC = annual percentage change, CI = confidence interval, SA-AKI = sepsis and acute kidney injury, U.S. = United States.

Disparities in SA-AKI-related mortality across U.S. census regions were observed over the 25-year period. All regions showed increasing AAMRs, with the South recording the highest absolute rate and the West the steepest growth (AAPC of 7.28% [95% CI: 4.69–9.94%]). Regional level trends and CI are detailed in: (Fig. 4, Table S7, Supplemental Digital Content 7) (Fig. S1, Supplemental Digital Content 10).

### 3.5. Sepsis and AKI-related AAMR stratified by urbanization

In metropolitan areas, AAMRs increased steadily from 3.55 in 1999 to 8.54 in 2020

**Figure 5.**
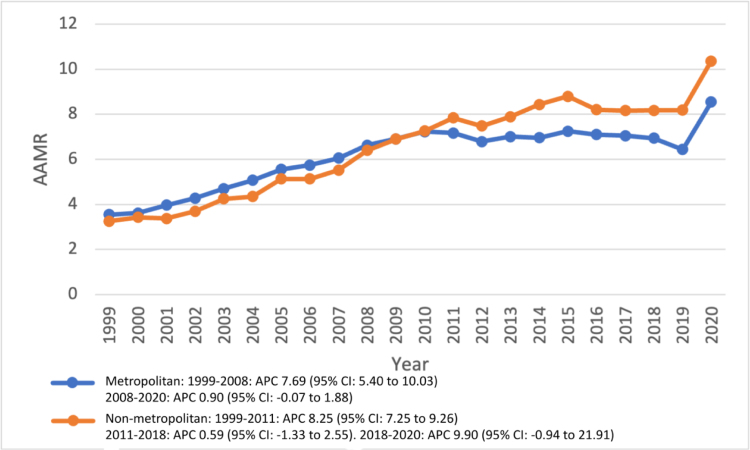
SA-AKI-related AAMRs per 100,000 stratified by urbanization in the U.S., between 1999 and 2023. AAMR = age-adjusted mortality rate, APC = annual percentage change, CI = confidence interval, SA-AKI = sepsis and acute kidney injury, U.S. = United States.

Similarly, nonmetropolitan areas also showed a consistent overall growth trend in AAMRs, with steady increases from 3.26 in 1999 to 7.84 in 2011 (APC: 8.25% [95% CI: 7.25–9.26%]), followed by a slowdown in growth and then increasing to 10.36 (10.04–10.68) between 2019 to 2020 (APC: 9.90% [95% CI: −0.94–21.91%]) (Fig. 5, Table S5, Supplemental Digital Content 5, Table S8, Supplemental Digital Content 8) (Fig. S1, Supplemental Digital Content 10).

### 3.6. Sepsis and AKI-related AAMR stratified by age group

Among young adults, AAMRs, despite showing a positive trend, remained very low and exhibited minimal change over 25 years. It increased from 0.36 to only 0.84 in 2018 (APC: 4.45% [95% CI: 3.89–5.02%]). Then, there was a sharp increase to 2.04 in 2021 (APC: 35.65% [95% CI: 18.28–55.58%]), followed by a decline to 1.62 in 2023 (APC: −6.28% [95% CI: −14.88–3.18%]).

Middle-aged adults initially showed a pattern similar to young adults, increasing from 1.75 in 1999 to 4.37 in 2015 (APC: 5.78% [95% CI: 5.06–6.50%]). Then, rates declined slightly to 4.43 in 2018, after a rise to 4.51 in a previous year, with a marked increase as AAMR grew to 10.44 by 2021 (APC: 36.56% [95% CI: 21.45–53.55%]). However, it decreased again to 8.46 in 2023 (APC: −5.99% [95% CI: −13.98–2.74%]).

**Figure 6.**
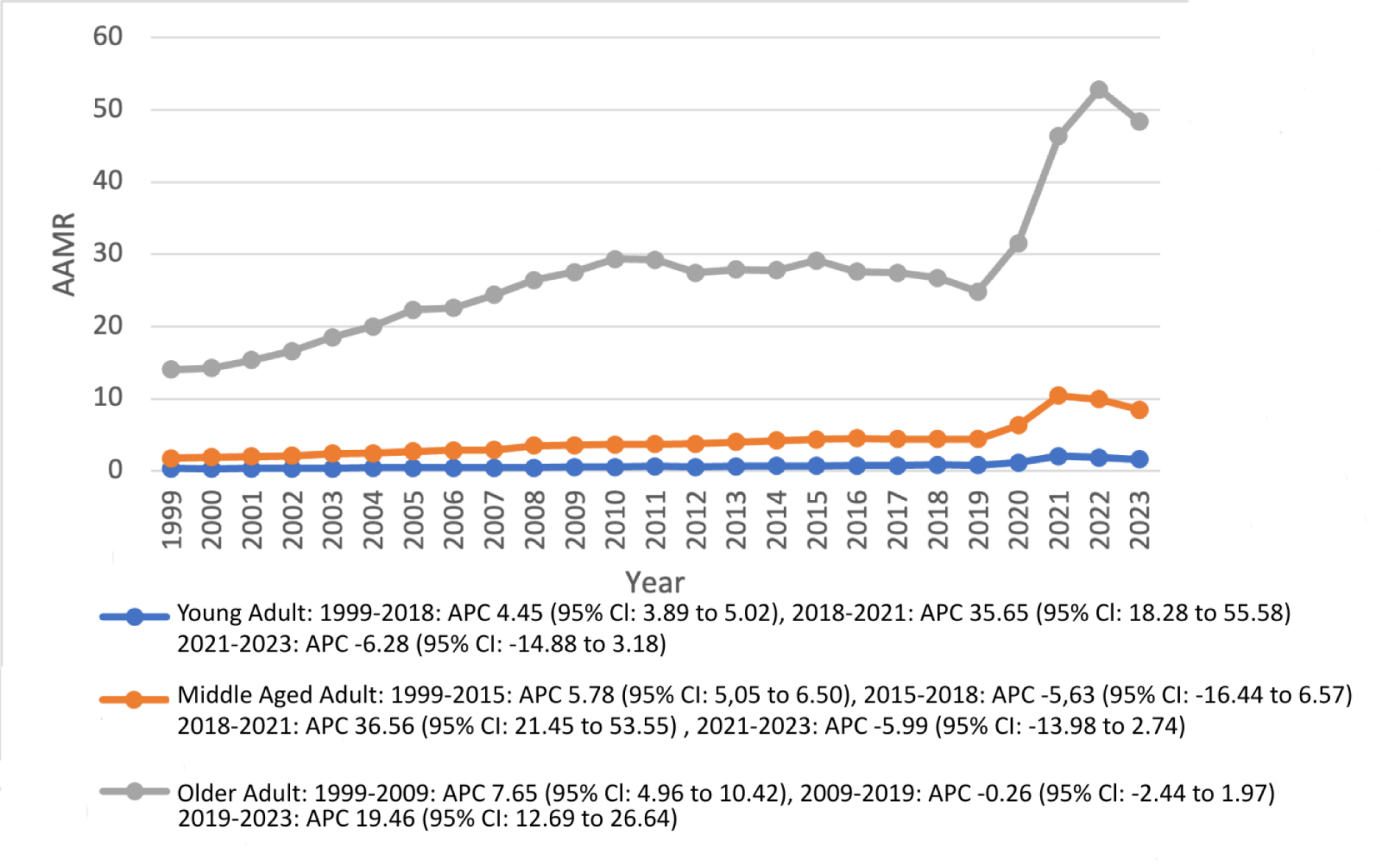
SA-AKI-related AAMRs per 100,000 stratified by age in the U.S., from 1999 to 2023. AAMR = age-adjusted mortality rate, APC = annual percentage change, CI = confidence interval, SA-AKI = sepsis and acute kidney injury, U.S. = United States.

In contrast to the previous groups, older adults showed the greatest variation in mortality rate trends. Starting with the highest initial AAMR of 14.04 in 1999, rates rose to 27.56 in 2009 (APC: 7.65% [95% CI: 4.96–10.42%]). Between 2009 and 2019, rates declined slightly each year, ending at 24.84. After 2019, the AAMR increased sharply, peaking at 52.84 in 2022 and ending at 48.38 in 2023 (APC: 19.46% [95% CI: 12.69–26.64%]) (Fig. 6, Table S1, Supplemental Digital Content 1, Table S8, Supplemental Digital Content 8) (Fig. S1, Supplemental Digital Content 10).

### 3.7. Sepsis and AKI-related AAMR stratified by state

**Figure 7.**
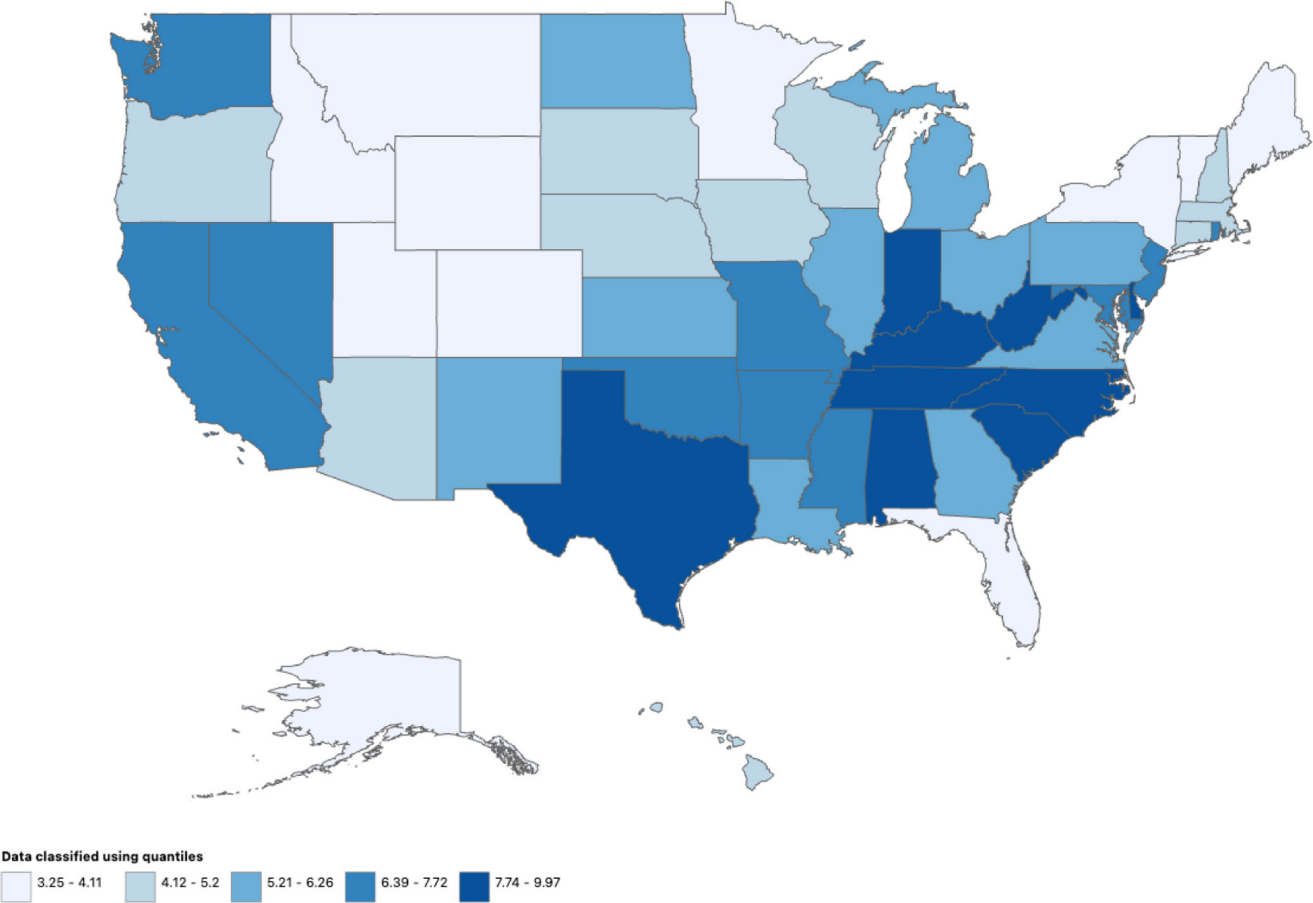
SA-AKI-related AAMRs per 100,000 stratified by State in the U.S., from 1999 to 2023. AAMR = age-adjusted mortality rate, SA-AKI = sepsis and acute kidney injury, U.S. = United States.

AAMR varied substantially across states between 1999 and 2019, ranging from 3.25 (95% CI: 2.90–3.61) in Vermont to 9.97 (95% CI: 9.86–10.09) in Texas. States in the highest percentile included Texas, the District of Columbia, South Carolina, Kentucky, and Delaware, whereas Vermont, Colorado, Montana, Maine, and New York had the lowest AAMRs (Fig. 7, Table S6, Supplemental Digital Content 6).

### 3.8. Sepsis and AKI: underlying causes of death

We assessed the top 15 underlying causes of death in patients with SA-AKI. Among these, septicemia was the leading underlying cause of death (66,555 deaths), followed by nephritis, nephrotic syndrome, and nephrosis (52,019 deaths), malignant neoplasms (41,500 deaths), diseases of the heart (28,252 deaths), and influenza and pneumonia (24,247 deaths). These findings suggest that SA-AKI-related mortality frequently occurred in the setting of substantial multisystem disease burden. The high prevalence of malignancy, cardiovascular disease, respiratory infections, and other severe underlying conditions indicates that many individuals represented medically complex, critically ill populations with multiple competing contributors to mortality (Table S9, Supplemental Digital Content 9).

## 4. Discussion

This 24-year analysis of CDC WONDER data reveals a significant and evolving burden of SA-AKI-related mortality in the U.S., with distinct temporal and demographic disparities. Across all stratifications (sex, race, census region, state, urbanization, and age) mortality rates exhibited a consistent pattern: a gradual increase through the early 2000s, a period of stabilization up to 2018, and a sharp resurgence in the years following. Males exhibited consistently higher mortality rates than females throughout the study period. Among racial groups, Black and American Indian or Alaska Native individuals experienced the highest mortality rates, while Hispanic and White populations generally had lower rates. Age-based analysis showed older adults (≥ 65 years) carried the greatest burden, accounting for nearly 75% of all deaths and exhibiting the highest AAMR. Geographically, the South recorded the highest mortality, while the West showed the steepest rate of increase over time. Rural (nonmetropolitan) areas experienced sharper mortality growth compared to urban centers, and wide disparities were observed at the state level, with Texas reporting the highest and Vermont the lowest AAMRs. These trends underscore the multifaceted and unequal impact of SA-AKI-related mortality across the U.S. population.

SA-AKI-related mortality exhibited a gradual upward trend from 1999 until approximately 2010, followed by a period of relative stabilization through 2018. However, from 2018 onward, the trend rose sharply, peaking during the COVID-19 pandemic in 2021, before declining rapidly through 2023. Although Joinpoint regression identified an inflection point beginning around 2018, annual mortality rates during 2018 to 2019 remained relatively stable prior to the substantial rise observed during the COVID-19 pandemic years. Sensitivity analyses excluding 2020 to 2023 demonstrated attenuation of the late-period increase, supporting the interpretation that pandemic-related healthcare disruptions and infection burden were major contributors to the sharp mortality acceleration observed in the primary analysis. The post-2018 spike is likely multifactorial, with COVID-19 acting as a major aggravating factor. The observed mortality increase during the pandemic period may partially reflect broader healthcare disruptions reported during COVID-19, including strained critical care capacity, delayed access to routine care, and interruptions in chronic disease management. Many individuals with known or undiagnosed kidney dysfunction faced delays or complete interruptions in care due to overwhelmed hospitals, lockdowns, and fear of viral exposure. Such disruptions may have contributed to worsening sepsis severity, delayed recognition of AKI, and poorer outcomes reported during the pandemic period. Su, Hua et al reported that Severe Acute Respiratory Syndrome Coronavirus 2 has been shown to directly infect renal tubular epithelial cells and podocytes, contributing to acute tubular injury, endothelial dysfunction, and thrombotic microangiopathy. These mechanisms may be a potential explanation for the wide clinical range of AKI incidence in COVID-19 patients, reported between 0.9% and 29% across different centers, particularly among those with comorbidities like diabetes and hypertension (HTN).^[15]^ These effects were particularly pronounced in racial minorities and rural populations, amplifying existing healthcare disparities. Together, these disruptions offer a plausible explanation for the marked rise in mortality during the pandemic period. The modest decline observed after 2021 may potentially reflect improvements in healthcare delivery, earlier recognition of clinical deterioration, and broader implementation of evidence-based critical care practices reported in prior studies.^[16]^ Greater adherence to Kidney Disease: Improving Global Outcomes (KDIGO) guidelines for AKI prevention and management,^[17]^ enhanced critical care practices, and earlier use of renal replacement therapies. Although these factors cannot be directly evaluated using the present dataset, they provide plausible contextual explanations for the observed temporal patterns. An additional consideration when interpreting long-term mortality trends is the potential influence of diagnostic drift over time. During the study period, evolving clinical definitions and increasing awareness of both sepsis and AKI may have altered diagnostic recognition and coding practices. The introduction of standardized KDIGO criteria for AKI in 2012 improved the uniformity and sensitivity of AKI diagnosis in clinical settings.^[18]^ Similarly, the Sepsis-3 consensus definition published in 2016 refined sepsis identification by emphasizing organ dysfunction and sequential organ failure assessment-based assessment.^[19]^ Previous studies have demonstrated that changes in sepsis definitions, coding practices, and administrative documentation can substantially influence epidemiologic estimates and reported mortality trends.^[20,21]^ Increased adoption of electronic health records, enhanced surveillance protocols, and greater clinician awareness may also have contributed to higher reporting of sepsis and AKI on death certificates over time. Consequently, part of the observed increase in SA-AKI-related mortality may reflect improved diagnostic ascertainment and coding practices rather than solely a true rise in disease burden.

We also report gender disparity, with males consistently exhibiting higher AAMRs than females across the study period. This disparity may be attributed to the immunomodulatory effects of sex hormones. Estrogen in females enhances immune responses, while testosterone in males is associated with immune suppression and pro-inflammatory activity, contributing to worse outcomes in septic and AKI states.^[22]^ Hormonal influences may also impair endothelial function and vascular regulation in males, increasing their susceptibility to organ failure.^[23]^ Behaviorally, men have higher rates of substance abuse involving nephrotoxic drugs such as alcohol, heroin, cocaine, and methamphetamines, which are known contributors to acute tubular necrosis, rhabdomyolysis, and sepsis-related infections like endocarditis.^[24,25]^ Furthermore, lower engagement in preventive healthcare and delayed care-seeking behavior among men likely contribute to more advanced disease at the time of presentation. However, improvements in-hospital care, early detection protocols, and broader implementation of standardized clinical guidelines aimed at better managing acute conditions like SA-AKI may have temporarily mitigated mortality across both sexes during the mid-2010s. In addition to sex-based disparities, racial and ethnic differences in SA-AKI-related mortality were striking and uneven.

Among all groups, NH American Indian or Alaska Native individuals experienced some of the highest and most fluctuating mortality rates. This is likely because many NH American Indian or Alaska Native individuals reside in rural and remote regions with limited access to advanced healthcare services, such as dialysis and critical care units.^[26]^ The underfunding of the Indian Health Service and a high burden of comorbidities like diabetes and HTN further compound their risk.^[27]^ The COVID-19 pandemic exacerbated these challenges, with NH American Indian or Alaska Native communities experiencing some of the highest infection and mortality rates in the U.S. due to delayed access to testing, treatment, and vaccines.^[28,29]^ Similarly, NH Black or African American individuals consistently exhibited higher AAMRs, which may be explained by long-standing structural racism,^[30]^ reduced access to preventive and specialty care, and a greater prevalence of sepsis-related comorbidities. In comparison, NH White individuals had lower AAMRs than NH American Indian or Alaska Native and NH Black populations but showed a steady upward trend over time. A key contributing factor may be the opioid epidemic,^[31,32]^ particularly in rural and suburban communities, which has led to increasing rates of drug-related infections, rhabdomyolysis, and AKI. Finally, Hispanic or Latino populations, historically considered to have a mortality advantage despite socioeconomic adversity (the “Hispanic paradox”),^[33]^ also experienced rising AAMRs. However, their rates remained lower than those observed in NH American Indian or Alaska Native and NH Black populations. Contributing factors may include limited health insurance coverage,^[34]^ language barriers, and increased exposure through frontline or essential work roles.

Geographic and urbanization-based disparities in SA-AKI-related mortality were evident across the U.S. By 2023, the South recorded the highest AAMR, while the West exhibited the steepest growth. At the state level, Southern states such as Texas and South Carolina consistently ranked in the top mortality percentiles, in contrast to Northeastern states like Vermont and New York, which reported among the lowest AAMRs. Similarly, when stratified by urbanization, nonmetropolitan (rural) areas showed consistently higher AAMRs and a sharper rate of increase than metropolitan regions, particularly in recent years. Rural communities, more prevalent in the South and West,^[35]^ often face critical limitations such as shortages of healthcare providers, limited specialty services like nephrology or critical care, fewer dialysis and intensive care unit facilities, and long distances to tertiary care.^[36]^ These barriers lead to delays in diagnosing and managing SA-AKI, both of which are time-sensitive, life-threatening conditions. Sepsis remains the leading cause of AKI, and previous research has shown persistently higher sepsis-related mortality in rural versus urban counties from 2010 to 2019.^[37]^ Limited access to timely nephrology consultation, known to improve AKI outcomes, further exacerbates rural-urban disparities, as ambulatory care use remains low in rural settings despite higher acute care utilization.^[38]^ Previous research has also shown that residents of nonmetropolitan areas report poorer physical and mental health, higher rates of comorbidities, and a greater prevalence of health-related risk factors for major causes of death. They are also more likely to be uninsured or underinsured, delay seeking care, and have lower educational attainment, all of which compound their vulnerability to preventable mortality.^[39]^

In contrast, metropolitan areas, particularly those in the Northeast, benefit from stronger healthcare infrastructure, higher ambulatory care utilization, and earlier adoption of quality improvement measures in SA-AKI management. These advantages support faster diagnosis, earlier nephrology consultation, and better chronic disease control, contributing to slower mortality increases and better outcomes. These findings collectively underscore the importance of regionally and structurally targeted healthcare reforms to reduce geographic disparities and improve access to life-saving interventions.

Age-based analysis of SA-AKI-related AAMRs demonstrated notable variation in both overall mortality and temporal trends across different age groups. Young adults consistently had the lowest AAMRs, and mortality remains relatively low due to greater physiological resilience, stronger immune responses, and fewer chronic conditions.^[40]^ However, the sharp increase may be attributed to under-recognition of sepsis in outpatient or emergency settings in younger patients, where severe illness is often less suspected. It is reported that among patients who develop sepsis, an estimated 8.2 to 20.8% experience a missed or delayed diagnosis.^[41]^ Moreover, limited healthcare engagement, delayed symptom reporting, and higher substance use (including nephrotoxic agents) in this group may lead to rapid deterioration and increased fatality once AKI or sepsis develops. Middle-aged adults often represent a transitional group, where the early onset of chronic illnesses such as diabetes, HTN, and obesity increases susceptibility to SA-AKI.^[42,43]^ This age group is frequently underscreened and may have poorer adherence to preventive care, increasing the risk of delayed diagnosis and more severe clinical presentations.^[44]^ Additionally, higher rates of occupational stress, substance use, and inconsistent healthcare engagement further exacerbate vulnerability in this cohort. In older adults, the high mortality burden can be attributed to a decline in immune function, diminished renal reserve, and the cumulative impact of comorbidities such as cardiovascular disease and chronic kidney disease.^[45]^ This population also faces a higher risk of iatrogenic harm due to polypharmacy and frequent exposure to nephrotoxic medications and invasive interventions.^[46–48]^ While older adults often have greater contact with healthcare services, they may still face barriers to timely specialist consultation, and their limited physiological reserve makes recovery from septic or renal insults particularly challenging.^[49]^

The underlying cause-of-death analysis further highlights the substantial comorbid and multisystem disease burden within the study population. In addition to septicemia and renal disease, malignancy, cardiovascular disease, respiratory infections, and other severe conditions were frequently documented as underlying causes of death. These findings suggest that many patients represented medically complex, critically ill populations in whom sepsis and AKI occurred within broader patterns of multiorgan dysfunction and competing comorbid illnesses. Consequently, the observed mortality trends likely reflect not only the burden of concurrent sepsis and AKI, but also the cumulative impact of severe chronic disease, critical illness, and systemic physiologic deterioration.

To address the rising burden of SA-AKI-related mortality, especially in underserved and rural communities, targeted interventions are essential. The American Society of Nephrology has initiated programs to raise awareness and promote early detection of AKI through multidisciplinary care models that can be adapted to resource-limited settings.^[35]^ In addition to acute triggers like sepsis and surgery, chronic conditions such as diabetes and HTN remain major contributors to AKI risk and disproportionately affect rural populations. Expanding access to preventive care, education, and chronic disease self-managementstrategies is critical for reducing mortality in these communities. Moreover, clinical evidence underscores that patients often exhibit early signs of deterioration hours before sepsis develops. Implementation of validated early warning tools, such as the modified early warning score, early warning scoring system, and national early warning score, offers a practical and evidence-based method for identifying high-risk patients.^[50]^ Incorporating these tools into routine clinical practice alongside community-based education and robust surveillance systems offers a promising path forward. Ultimately, a multifaceted, equity-focused approach that integrates acute care, chronic disease management, and early detection is key to reversing current trends and improving SA-AKI outcomes nationwide.

## 5. Limitations

This study has several limitations.

This study relied exclusively on ICD-10 coding from death certificate data, which may be subject to coding inaccuracies, underreporting, and interinstitutional variability in documentation practices. The coexistence of sepsis and AKI codes likely represents a clinically heterogeneous population of critically ill patients rather than exclusively primary sepsis-induced AKI cases. Furthermore, the CDC WONDER database lacks detailed clinical information, including baseline renal function, serum creatinine trends, urine output criteria, timing of organ dysfunction, illness severity, and KDIGO staging. Consequently, AKI could not be clinically validated beyond physician-reported ICD coding, limiting causal interpretation and precise phenotypic characterization of SA-AKI-related mortality. Additionally, urbanization data were only available through 2020, and similarly, state-level data were only collected until 2019 to avoid the influence of COVID-19 deaths, which limited the ability to analyze recent geographic trends. Moreover, death certificate-based analyses are inherently subject to coding inaccuracies, underreporting, and variability in physician documentation and certification practices across institutions and regions. Because ICD-10 codes were derived from multiple cause-of-death fields, the coexistence of sepsis and AKI on the same death certificate does not confirm a temporal or causal relationship between the 2 conditions. Therefore, our findings should be interpreted as mortality involving concurrent sepsis and AKI rather than definitively causal SA-AKI events. Furthermore, the database lacked information regarding healthcare access, hospital capacity, treatment practices, illness severity, and longitudinal clinical management, limiting the ability to directly evaluate mechanisms underlying observed mortality trends. Additionally, evolving diagnostic criteria and coding practices for sepsis and AKI over the 24-year study period may have influenced temporal mortality trends. Changes in clinical recognition, implementation of KDIGO and Sepsis-3 frameworks, and increased documentation sensitivity may have contributed to temporal variations in coding and reporting. Lastly, restriction of the cohort to individuals aged ≥ 25 years may limit generalizability to younger adult populations aged 18 to 24 years. However, this approach was necessary to maintain consistency with the standardized age-group structure used for CDC WONDER age-adjusted mortality calculations while preserving an exclusively adult cohort.

## 6. Conclusion

Mortality involving concurrent sepsis and AKI documented on death certificates increased substantially in the U.S. from 1999 to 2023, with marked demographic and geographic disparities. The largest increases were observed during the pandemic period, although the observational nature of the data precludes causal inference regarding underlying mechanisms. Higher mortality rates were observed among older adults, males, racial minorities, and residents of nonmetropolitan regions. These findings highlight the importance of improving equitable access to preventive care, early recognition systems, and evidence-based management strategies for populations at elevated risk of severe sepsis and kidney injury.

## Author contributions

**Conceptualization:** Rahul Balach.

**Formal analysis:** Rahul Balach.

**Methodology:** Muhammad Ahsan.

**Project administration:** Hasibullah Aminpoor.

**Software:** Muhammad Ahsan.

**Supervision:** Muhammad Ibrahim, Adnan Safi, Saad Ahmed Waqas.

**Validation:** Muhammad Ibrahim, Adnan Safi.

**Writing – original draft:** Shahtaj Tariq, Muhammad Taha Nizami, Shafaq Jawed.

**Writing – review & editing:** Muhammad Ibrahim, Saad Ahmed Waqas.




















