# Supplementary material for: Sepsis and acute kidney injury-related mortality in the U.S.: National trends and disparities (1999–2023)
Source: Medicine (Baltimore). 2026 Jun 26;105(26):e49495. doi: 10.1097/MD.0000000000049495 (PMC13313787; doi:10.1097/MD.0000000000049495)
Supplement: Supplementary file 1 [file medi-105-e49495-s001.docx]

| **Place of death** | **Number of Deaths** |
| --- | --- |
| **Medical Facility** | 380436 |
| **Decedent's home** | 6511 |
| **Hospice facility** | 10145 |
| **Nursing home/long term care** | 11827 |

**Supplementary Table 1:**Sepsis and AKI associated mortality among United States adults with stratified by place of death from 1999 to 2023.
