# Supplementary material for: Sepsis and acute kidney injury-related mortality in the U.S.: National trends and disparities (1999–2023)
Source: Medicine (Baltimore). 2026 Jun 26;105(26):e49495. doi: 10.1097/MD.0000000000049495 (PMC13313787; doi:10.1097/MD.0000000000049495)
Supplement: Supplementary file 2 [file medi-105-e49495-s002.docx]

| **Age-Adjusted Rate (95% CI)** | | | |
| --- | --- | --- | --- |
|  | **Young Adult** | **Middle Aged** | **Older Adult** |
| **1999** | 0.36 (0.32–0.41) | 1.75 (1.64–1.86) | 14.04 (13.65–14.44) |
| **2000** | 0.32 (0.28–0.35) | 1.91 (1.8–2.02) | 14.23 (13.83–14.63) |
| **2001** | 0.36 (0.32–0.41) | 2.01 (1.9–2.12) | 15.35 (14.94–15.76) |
| **2002** | 0.36 (0.32–0.4) | 2.11 (2–2.22) | 16.6 (16.18–17.03) |
| **2003** | 0.36 (0.32–0.4) | 2.39 (2.27–2.5) | 18.51 (18.07–18.96) |
| **2004** | 0.42 (0.37–0.46) | 2.43 (2.31–2.54) | 20.02 (19.56–20.48) |
| **2005** | 0.42 (0.37–0.46) | 2.7 (2.59–2.82) | 22.3 (21.82–22.78) |
| **2006** | 0.47 (0.43–0.52) | 2.88 (2.76–3) | 22.56 (22.08–23.03) |
| **2007** | 0.42 (0.38–0.46) | 2.92 (2.8–3.04) | 24.37 (23.88–24.87) |
| **2008** | 0.47 (0.43–0.52) | 3.52 (3.39–3.65) | 26.44 (25.93–26.95) |
| **2009** | 0.57 (0.52–0.62) | 3.58 (3.45–3.71) | 27.56 (27.04–28.07) |
| **2010** | 0.52 (0.47–0.57) | 3.66 (3.53–3.79) | 29.34 (28.81–29.87) |
| **2011** | 0.63 (0.57–0.68) | 3.7 (3.57–3.83) | 29.24 (28.72–29.76) |
| **2012** | 0.57 (0.52–0.63) | 3.78 (3.65–3.91) | 27.42 (26.93–27.92) |
| **2013** | 0.63 (0.57–0.68) | 4.01 (3.88–4.15) | 27.91 (27.41–28.41) |
| **2014** | 0.68 (0.62–0.74) | 4.19 (4.06–4.33) | 27.81 (27.32–28.3) |
| **2015** | 0.68 (0.62–0.74) | 4.37 (4.23–4.51) | 29.15 (28.66–29.65) |
| **2016** | 0.73 (0.67–0.79) | 4.51 (4.37–4.65) | 27.61 (27.13–28.08) |
| **2017** | 0.73 (0.67–0.79) | 4.41 (4.27–4.55) | 27.45 (26.98–27.92) |
| **2018** | 0.84 (0.77–0.9) | 4.43 (4.29–4.57) | 26.73 (26.28–27.19) |
| **2019** | 0.78 (0.72–0.84) | 4.39 (4.25–4.53) | 24.84 (24.41–25.28) |
| **2020** | 1.15 (1.07–1.22) | 6.3 (6.13–6.47) | 31.53 (31.06–32.01) |
| **2021** | 2.04 (1.94–2.13) | 10.44 (10.22–10.65) | 46.34 (45.75–46.93) |
| **2022** | 1.87 (1.78–1.97) | 9.92 (9.71–10.13) | 52.84 (52.23–53.45) |
| **2023** | 1.62 (1.53–1.7) | 8.46 (8.27–8.66) | 48.39 (47.81–48.98) |

**Supplementary Table 2:**Sepsis and AKI associated AAMR per 100,000 stratified by Age Group in the United States from 1999-2023
