# Supplementary material for: Sepsis and acute kidney injury-related mortality in the U.S.: National trends and disparities (1999–2023)
Source: Medicine (Baltimore). 2026 Jun 26;105(26):e49495. doi: 10.1097/MD.0000000000049495 (PMC13313787; doi:10.1097/MD.0000000000049495)
Supplement: Supplementary file 3 [file medi-105-e49495-s003.docx]

| **Age-Adjusted Rate (95% CI)** | | | |
| --- | --- | --- | --- |
| **Year** | **Overall** | **Male** | **Female** |
| **1999** | 3.51 (3.43–3.6) | 4.62 (4.46–4.78) | 2.76 (2.66–2.86) |
| **2000** | 3.58 (3.5–3.67) | 4.57 (4.41–4.73) | 2.93 (2.83–3.04) |
| **2001** | 3.86 (3.77–3.95) | 4.88 (4.71–5.04) | 3.18 (3.07–3.28) |
| **2002** | 4.14 (4.04–4.23) | 5.3 (5.13–5.47) | 3.39 (3.28–3.5) |
| **2003** | 4.61 (4.51–4.7) | 5.7 (5.53–5.88) | 3.9 (3.78–4.02) |
| **2004** | 4.94 (4.84–5.04) | 6.07 (5.89–6.25) | 4.17 (4.05–4.29) |
| **2005** | 5.48 (5.38–5.58) | 6.7 (6.52–6.89) | 4.63 (4.5–4.75) |
| **2006** | 5.62 (5.51–5.72) | 6.87 (6.69–7.06) | 4.77 (4.65–4.9) |
| **2007** | 5.96 (5.85–6.07) | 7.24 (7.05–7.42) | 5.04 (4.91–5.17) |
| **2008** | 6.59 (6.48–6.7) | 7.82 (7.63–8.01) | 5.72 (5.58–5.85) |
| **2009** | 6.88 (6.77–6.99) | 8.26 (8.06–8.45) | 5.91 (5.77–6.05) |
| **2010** | 7.23 (7.11–7.34) | 8.59 (8.39–8.78) | 6.27 (6.13–6.41) |
| **2011** | 7.27 (7.16–7.39) | 8.6 (8.41–8.8) | 6.31 (6.17–6.45) |
| **2012** | 6.92 (6.81–7.03) | 8.13 (7.95–8.32) | 5.98 (5.84–6.11) |
| **2013** | 7.12 (7.01–7.23) | 8.26 (8.08–8.44) | 6.3 (6.16–6.44) |
| **2014** | 7.19 (7.08–7.3) | 8.45 (8.27–8.63) | 6.3 (6.16–6.44) |
| **2015** | 7.51 (7.4–7.62) | 8.71 (8.53–8.89) | 6.59 (6.45–6.73) |
| **2016** | 7.28 (7.17–7.39) | 8.57 (8.39–8.75) | 6.27 (6.14–6.4) |
| **2017** | 7.21 (7.11–7.32) | 8.53 (8.36–8.71) | 6.22 (6.09–6.36) |
| **2018** | 7.13 (7.03–7.24) | 8.42 (8.24–8.59) | 6.13 (6–6.26) |
| **2019** | 6.72 (6.62–6.83) | 7.95 (7.78–8.11) | 5.8 (5.67–5.92) |
| **2020** | 8.85 (8.74–8.97) | 10.85 (10.66–11.04) | 7.21 (7.07–7.35) |
| **2021** | 13.58 (13.44–13.72) | 16.66 (16.42–16.89) | 11.03 (10.86–11.21) |
| **2022** | 14.6 (14.45–14.74) | 17.42 (17.18–17.67) | 12.44 (12.26–12.62) |
| **2023** | 13.11 (12.97–13.25) | 15.22 (15–15.44) | 11.45 (11.28–11.63) |

**Supplementary Table 3:** Overall and sex-stratified Sepsis and AKI associated AAMR per 100,000 in the United States from 1999-2023.
