# Supplementary material for: Sepsis and acute kidney injury-related mortality in the U.S.: National trends and disparities (1999–2023)
Source: Medicine (Baltimore). 2026 Jun 26;105(26):e49495. doi: 10.1097/MD.0000000000049495 (PMC13313787; doi:10.1097/MD.0000000000049495)
Supplement: Supplementary file 4 [file medi-105-e49495-s004.docx]

| **Age-Adjusted Rate (95% CI)** | | | | |
| --- | --- | --- | --- | --- |
| **Year** | **NH American Indian or Alaska Native** | **NH Black or African American** | **NH White** | **Hispanic or Latino** |
| **1999** | 4.89 (3.34–6.9) | 6.97 (6.54–7.4) | 3.1 (3.01–3.19) | 3.77 (3.35–4.2) |
| **2000** | 3.61 (2.4–5.22) | 7.15 (6.72–7.58) | 3.21 (3.11–3.3) | 3.73 (3.31–4.14) |
| **2001** | 4.53 (3.12–6.37) | 7.77 (7.32–8.21) | 3.46 (3.37–3.56) | 3.88 (3.48–4.29) |
| **2002** | 4.77 (3.39–6.52) | 8.13 (7.67–8.58) | 3.7 (3.61–3.8) | 4.6 (4.17–5.03) |
| **2003** | 4.84 (3.46–6.59) | 8.75 (8.29–9.22) | 4.21 (4.11–4.32) | 4.55 (4.13–4.97) |
| **2004** | 4.79 (3.44–6.5) | 9.46 (8.98–9.95) | 4.5 (4.39–4.6) | 5.13 (4.7–5.56) |
| **2005** | 6.03 (4.46–7.97) | 9.88 (9.39–10.37) | 5.05 (4.94–5.16) | 5.79 (5.35–6.24) |
| **2006** | 5.6 (4.14–7.4) | 10.13 (9.64–10.62) | 5.15 (5.04–5.26) | 5.44 (5.02–5.86) |
| **2007** | 9.78 (7.81–12.09) | 10.48 (9.98–10.97) | 5.51 (5.39–5.62) | 6.26 (5.81–6.7) |
| **2008** | 8.51 (6.75–10.59) | 10.99 (10.49–11.48) | 6.17 (6.05–6.29) | 6.28 (5.84–6.72) |
| **2009** | 9.46 (7.57–11.35) | 10.96 (10.47–11.45) | 6.47 (6.35–6.59) | 6.71 (6.28–7.14) |
| **2010** | 9.83 (7.9–11.76) | 11.26 (10.77–11.76) | 6.8 (6.68–6.93) | 7.27 (6.82–7.71) |
| **2011** | 10.13 (8.24–12.02) | 10.69 (10.22–11.16) | 6.95 (6.82–7.07) | 7.05 (6.62–7.47) |
| **2012** | 11.72 (9.7–13.75) | 9.76 (9.32–10.2) | 6.63 (6.51–6.75) | 6.42 (6.03–6.81) |
| **2013** | 10.47 (8.65–12.3) | 10.48 (10.03–10.93) | 6.83 (6.71–6.95) | 6.92 (6.53–7.31) |
| **2014** | 12.1 (10.17–14.02) | 9.67 (9.25–10.1) | 6.94 (6.82–7.06) | 7.18 (6.8–7.57) |
| **2015** | 11.8 (9.98–13.63) | 10.19 (9.76–10.62) | 7.29 (7.16–7.41) | 7.45 (7.06–7.83) |
| **2016** | 10.8 (9.09–12.51) | 9.63 (9.22–10.03) | 7.03 (6.91–7.16) | 7.39 (7.02–7.76) |
| **2017** | 11.52 (9.78–13.27) | 9.5 (9.11–9.9) | 6.89 (6.77–7.01) | 8.11 (7.73–8.48) |
| **2018** | 10.24 (8.64–11.85) | 9.44 (9.05–9.83) | 6.88 (6.76–7) | 7.53 (7.18–7.89) |
| **2019** | 10.64 (9.03–12.26) | 9.12 (8.75–9.5) | 6.47 (6.36–6.59) | 6.85 (6.52–7.18) |
| **2020** | 16.61 (14.66–18.57) | 13.48 (13.03–13.93) | 7.78 (7.65–7.9) | 11.93 (11.51–12.35) |
| **2021** | 24.54 (22.07–27.02) | 19.12 (18.58–19.67) | 12.63 (12.47–12.8) | 15.92 (15.45–16.4) |
| **2022** | 24.21 (21.75–26.66) | 20.7 (20.14–21.26) | 14.27 (14.09–14.44) | 13.95 (13.5–14.39) |
| **2023** | 19.96 (17.77–22.15) | 18.03 (17.51–18.54) | 12.96 (12.79–13.12) | 12.01 (11.6–12.42) |

**Supplementary Table 4:** Sepsis and AKI-associated AAMR per 100,000 stratified by Race in the United States from 1999-2023
