# Supplementary material for: Sepsis and acute kidney injury-related mortality in the U.S.: National trends and disparities (1999–2023)
Source: Medicine (Baltimore). 2026 Jun 26;105(26):e49495. doi: 10.1097/MD.0000000000049495 (PMC13313787; doi:10.1097/MD.0000000000049495)
Supplement: Supplementary file 5 [file medi-105-e49495-s005.docx]

| **Age-Adjusted Rate (95% CI)** | | |
| --- | --- | --- |
| **Year** | **Metropolitan** | **Non-metropolitan** |
| **1999** | 3.55 (3.45–3.65) | 3.26 (3.06–3.45) |
| **2000** | 3.62 (3.52–3.71) | 3.43 (3.23–3.63) |
| **2001** | 3.97 (3.87–4.08) | 3.37 (3.18–3.57) |
| **2002** | 4.28 (4.18–4.39) | 3.7 (3.5–3.91) |
| **2003** | 4.7 (4.59–4.81) | 4.25 (4.03–4.47) |
| **2004** | 5.07 (4.96–5.18) | 4.35 (4.13–4.57) |
| **2005** | 5.55 (5.43–5.66) | 5.13 (4.89–5.36) |
| **2006** | 5.74 (5.62–5.86) | 5.13 (4.9–5.37) |
| **2007** | 6.05 (5.93–6.17) | 5.52 (5.27–5.76) |
| **2008** | 6.63 (6.51–6.76) | 6.4 (6.14–6.66) |
| **2009** | 6.91 (6.78–7.03) | 6.9 (6.63–7.17) |
| **2010** | 7.23 (7.1–7.35) | 7.26 (6.99–7.54) |
| **2011** | 7.17 (7.05–7.3) | 7.84 (7.55–8.12) |
| **2012** | 6.79 (6.67–6.91) | 7.48 (7.21–7.76) |
| **2013** | 7.01 (6.89–7.13) | 7.89 (7.61–8.17) |
| **2014** | 6.96 (6.84–7.08) | 8.43 (8.14–8.72) |
| **2015** | 7.25 (7.13–7.37) | 8.79 (8.5–9.09) |
| **2016** | 7.1 (6.98–7.21) | 8.2 (7.91–8.48) |
| **2017** | 7.05 (6.93–7.16) | 8.16 (7.88–8.44) |
| **2018** | 6.94 (6.83–7.05) | 8.17 (7.88–8.45) |
| **2019** | 6.44 (6.34–6.55) | 8.18 (7.9–8.46) |
| **2020** | 8.54 (8.42–8.67) | 10.36 (10.04–10.68) |

**Supplementary Table 5:** Sepsis and AKI-associated AAMR per 100,000 stratified by urban-rural classification in the United States from 1999 to 2020.
