# Supplementary material for: Sepsis and acute kidney injury-related mortality in the U.S.: National trends and disparities (1999–2023)
Source: Medicine (Baltimore). 2026 Jun 26;105(26):e49495. doi: 10.1097/MD.0000000000049495 (PMC13313787; doi:10.1097/MD.0000000000049495)
Supplement: Supplementary file 6 [file medi-105-e49495-s006.docx]

| **State** | **Age-Adjusted Rate (95% CI)** |
| --- | --- |
| **Alabama** | 7.77 (7.56–7.97) |
| **Alaska** | 3.83 (3.32–4.34) |
| **Arizona** | 4.89 (4.75–5.03) |
| **Arkansas** | 7.72 (7.46–7.98) |
| **California** | 7.38 (7.31–7.46) |
| **Colorado** | 3.47 (3.33–3.62) |
| **Connecticut** | 4.12 (3.96–4.29) |
| **Delaware** | 8.31 (7.82–8.79) |
| **District of Columbia** | 9.47 (8.79–10.15) |
| **Florida** | 4.11 (4.04–4.18) |
| **Georgia** | 5.7 (5.56–5.84) |
| **Hawaii** | 5.04 (4.74–5.34) |
| **Idaho** | 4 (3.73–4.27) |
| **Illinois** | 5.43 (5.32–5.54) |
| **Indiana** | 7.86 (7.68–8.04) |
| **Iowa** | 4.4 (4.21–4.58) |
| **Kansas** | 5.21 (4.99–5.42) |
| **Kentucky** | 9.17 (8.93–9.41) |
| **Louisiana** | 6.26 (6.07–6.46) |
| **Maine** | 3.75 (3.5–4) |
| **Maryland** | 7.47 (7.28–7.66) |
| **Massachusetts** | 5.2 (5.06–5.34) |
| **Michigan** | 5.29 (5.17–5.41) |
| **Minnesota** | 4.05 (3.9–4.19) |
| **Mississippi** | 7.44 (7.17–7.7) |
| **Missouri** | 6.39 (6.22–6.55) |
| **Montana** | 3.56 (3.26–3.85) |
| **Nebraska** | 4.79 (4.54–5.05) |
| **Nevada** | 6.49 (6.21–6.77) |
| **New Hampshire** | 4.62 (4.32–4.92) |
| **New Jersey** | 6.78 (6.64–6.92) |
| **New Mexico** | 5.47 (5.2–5.74) |
| **New York** | 3.78 (3.71–3.85) |
| **North Carolina** | 7.74 (7.58–7.89) |
| **North Dakota** | 6.04 (5.57–6.5) |
| **Ohio** | 6.17 (6.06–6.29) |
| **Oklahoma** | 7.13 (6.91–7.36) |
| **Oregon** | 4.19 (4.03–4.36) |
| **Pennsylvania** | 5.75 (5.65–5.85) |
| **Rhode Island** | 7.51 (7.1–7.91) |
| **South Carolina** | 9.24 (9.01–9.47) |
| **South Dakota** | 4.67 (4.29–5.04) |
| **Tennessee** | 8.07 (7.88–8.25) |
| **Texas** | 9.97 (9.86–10.09) |
| **Utah** | 3.78 (3.55–4.01) |
| **Vermont** | 3.25 (2.9–3.61) |
| **Virginia** | 6.08 (5.93–6.22) |
| **Washington** | 6.52 (6.36–6.69) |
| **West Virginia** | 7.76 (7.45–8.07) |
| **Wisconsin** | 4.65 (4.51–4.8) |
| **Wyoming** | 4.05 (3.6–4.5) |

**Supplementary Table 6:** Sepsis and AKI associated AAMR per 100,000 stratified by state in the United States from 1999-2020
