# Supplementary material for: Sepsis and acute kidney injury-related mortality in the U.S.: National trends and disparities (1999–2023)
Source: Medicine (Baltimore). 2026 Jun 26;105(26):e49495. doi: 10.1097/MD.0000000000049495 (PMC13313787; doi:10.1097/MD.0000000000049495)
Supplement: Supplementary file 7 [file medi-105-e49495-s007.docx]

| **Census Reigon** | **Year** | **Age-Adjusted Rate (95% CI)** |
| --- | --- | --- |
| Census Region 1: Northeast | 1999 | 3.72 (3.53–3.92) |
| Census Region 1: Northeast | 2000 | 3.88 (3.68–4.08) |
| Census Region 1: Northeast | 2001 | 4.15 (3.95–4.36) |
| Census Region 1: Northeast | 2002 | 4.3 (4.1–4.51) |
| Census Region 1: Northeast | 2003 | 4.49 (4.28–4.7) |
| Census Region 1: Northeast | 2004 | 4.86 (4.64–5.07) |
| Census Region 1: Northeast | 2005 | 5.02 (4.8–5.24) |
| Census Region 1: Northeast | 2006 | 5.01 (4.79–5.23) |
| Census Region 1: Northeast | 2007 | 5.38 (5.16–5.61) |
| Census Region 1: Northeast | 2008 | 5.71 (5.48–5.94) |
| Census Region 1: Northeast | 2009 | 5.62 (5.39–5.84) |
| Census Region 1: Northeast | 2010 | 6.01 (5.77–6.24) |
| Census Region 1: Northeast | 2011 | 6.01 (5.78–6.24) |
| Census Region 1: Northeast | 2012 | 5.45 (5.23–5.67) |
| Census Region 1: Northeast | 2013 | 5.56 (5.34–5.78) |
| Census Region 1: Northeast | 2014 | 5.18 (4.97–5.4) |
| Census Region 1: Northeast | 2015 | 5.14 (4.93–5.35) |
| Census Region 1: Northeast | 2016 | 5.1 (4.89–5.31) |
| Census Region 1: Northeast | 2017 | 4.73 (4.53–4.93) |
| Census Region 1: Northeast | 2018 | 4.95 (4.75–5.16) |
| Census Region 1: Northeast | 2019 | 4.6 (4.41–4.8) |
| Census Region 1: Northeast | 2020 | 6.13 (5.91–6.36) |
| Census Region 1: Northeast | 2021 | 8.75 (8.48–9.01) |
| Census Region 1: Northeast | 2022 | 10.69 (10.4–10.98) |
| Census Region 1: Northeast | 2023 | 9.73 (9.45–10) |
| Census Region 2: Midwest | 1999 | 3.2 (3.03–3.37) |
| Census Region 2: Midwest | 2000 | 3.13 (2.96–3.3) |
| Census Region 2: Midwest | 2001 | 3.54 (3.36–3.72) |
| Census Region 2: Midwest | 2002 | 3.73 (3.55–3.91) |
| Census Region 2: Midwest | 2003 | 4.24 (4.04–4.43) |
| Census Region 2: Midwest | 2004 | 4.41 (4.22–4.61) |
| Census Region 2: Midwest | 2005 | 5.22 (5.01–5.43) |
| Census Region 2: Midwest | 2006 | 5.07 (4.87–5.28) |
| Census Region 2: Midwest | 2007 | 5.6 (5.38–5.82) |
| Census Region 2: Midwest | 2008 | 6.16 (5.93–6.38) |
| Census Region 2: Midwest | 2009 | 6.25 (6.02–6.47) |
| Census Region 2: Midwest | 2010 | 6.47 (6.24–6.7) |
| Census Region 2: Midwest | 2011 | 6.77 (6.54–7) |
| Census Region 2: Midwest | 2012 | 6.32 (6.09–6.54) |
| Census Region 2: Midwest | 2013 | 6.52 (6.3–6.75) |
| Census Region 2: Midwest | 2014 | 6.64 (6.42–6.87) |
| Census Region 2: Midwest | 2015 | 6.76 (6.53–6.98) |
| Census Region 2: Midwest | 2016 | 6.69 (6.47–6.92) |
| Census Region 2: Midwest | 2017 | 6.45 (6.23–6.67) |
| Census Region 2: Midwest | 2018 | 6.25 (6.04–6.46) |
| Census Region 2: Midwest | 2019 | 6.2 (5.99–6.41) |
| Census Region 2: Midwest | 2020 | 7.86 (7.63–8.1) |
| Census Region 2: Midwest | 2021 | 11.29 (11–11.58) |
| Census Region 2: Midwest | 2022 | 13.29 (12.98–13.59) |
| Census Region 2: Midwest | 2023 | 12.03 (11.74–12.32) |
| Census Region 3: South | 1999 | 3.84 (3.69–3.99) |
| Census Region 3: South | 2000 | 4.06 (3.9–4.22) |
| Census Region 3: South | 2001 | 4.29 (4.13–4.45) |
| Census Region 3: South | 2002 | 4.59 (4.43–4.76) |
| Census Region 3: South | 2003 | 5.27 (5.09–5.44) |
| Census Region 3: South | 2004 | 5.43 (5.25–5.61) |
| Census Region 3: South | 2005 | 6.27 (6.08–6.46) |
| Census Region 3: South | 2006 | 6.41 (6.22–6.6) |
| Census Region 3: South | 2007 | 6.74 (6.55–6.93) |
| Census Region 3: South | 2008 | 7.52 (7.32–7.72) |
| Census Region 3: South | 2009 | 7.99 (7.78–8.19) |
| Census Region 3: South | 2010 | 8.4 (8.19–8.61) |
| Census Region 3: South | 2011 | 8.11 (7.91–8.31) |
| Census Region 3: South | 2012 | 7.92 (7.73–8.12) |
| Census Region 3: South | 2013 | 8.28 (8.09–8.48) |
| Census Region 3: South | 2014 | 8.46 (8.26–8.66) |
| Census Region 3: South | 2015 | 8.89 (8.69–9.09) |
| Census Region 3: South | 2016 | 8.43 (8.24–8.62) |
| Census Region 3: South | 2017 | 8.48 (8.29–8.67) |
| Census Region 3: South | 2018 | 8.56 (8.37–8.74) |
| Census Region 3: South | 2019 | 7.84 (7.67–8.02) |
| Census Region 3: South | 2020 | 10.58 (10.38–10.78) |
| Census Region 3: South | 2021 | 16.55 (16.29–16.81) |
| Census Region 3: South | 2022 | 17.29 (17.04–17.55) |
| Census Region 3: South | 2023 | 15.13 (14.89–15.37) |
| Census Region 4: West | 1999 | 3.03 (2.85–3.22) |
| Census Region 4: West | 2000 | 2.98 (2.8–3.16) |
| Census Region 4: West | 2001 | 3.14 (2.96–3.33) |
| Census Region 4: West | 2002 | 3.68 (3.48–3.87) |
| Census Region 4: West | 2003 | 4.04 (3.84–4.24) |
| Census Region 4: West | 2004 | 4.73 (4.51–4.95) |
| Census Region 4: West | 2005 | 4.84 (4.62–5.05) |
| Census Region 4: West | 2006 | 5.48 (5.25–5.7) |
| Census Region 4: West | 2007 | 5.58 (5.35–5.81) |
| Census Region 4: West | 2008 | 6.31 (6.07–6.55) |
| Census Region 4: West | 2009 | 6.9 (6.65–7.14) |
| Census Region 4: West | 2010 | 7.15 (6.9–7.39) |
| Census Region 4: West | 2011 | 7.49 (7.24–7.74) |
| Census Region 4: West | 2012 | 7.05 (6.82–7.29) |
| Census Region 4: West | 2013 | 7.21 (6.97–7.45) |
| Census Region 4: West | 2014 | 7.38 (7.14–7.62) |
| Census Region 4: West | 2015 | 7.83 (7.59–8.07) |
| Census Region 4: West | 2016 | 7.63 (7.39–7.86) |
| Census Region 4: West | 2017 | 7.87 (7.63–8.1) |
| Census Region 4: West | 2018 | 7.34 (7.11–7.56) |
| Census Region 4: West | 2019 | 7.12 (6.9–7.34) |
| Census Region 4: West | 2020 | 8.96 (8.72–9.2) |
| Census Region 4: West | 2021 | 14.65 (14.34–14.97) |
| Census Region 4: West | 2022 | 14.44 (14.13–14.74) |
| Census Region 4: West | 2023 | 13.5 (13.21–13.8) |

**Supplementary Table 7:**Sepsis and AKI-associated AAMR per 100,000 stratified by census region in the United States from 1999-2023.
