# Supplementary material for: Sepsis and acute kidney injury-related mortality in the U.S.: National trends and disparities (1999–2023)
Source: Medicine (Baltimore). 2026 Jun 26;105(26):e49495. doi: 10.1097/MD.0000000000049495 (PMC13313787; doi:10.1097/MD.0000000000049495)
Supplement: Supplementary file 8 [file medi-105-e49495-s008.docx]

| **Interval of years** | **APC (95% CI)** |
| --- | --- |
| **Overall** | |
| 1999–2010 | 7.05 (3.87 to 10.32) |
| 2010–2018 | -0.43 (-4.90 to 4.24) |
| 2018–2023 | 16.93 (10.01 to 24.29) |
| **Male** | |
| 1999–2010 | 6.40 (5.83 to 6.97) |
| 2010–2018 | -1.49 (-2.27 to -0.71) |
| 2018–2021 | 26.77 (20.32 to 33.57) |
| 2021–2023 | 0.30 (-3.47 to 4.21) |
| **Female** | |
| 1999–2009 | 8.33 (5.46 to 11.28) |
| 2009–2019 | 0.49 (-1.86 to 2.89) |
| 2019–2023 | 20.27 (12.95 to 28.07) |
| **NH American Indian or Alaska Native** | |
| 1999–2014 | 8.08 (6.09 to 10.12) |
| 2014–2018 | -7.40 (-18.87 to 5.69) |
| 2018–2021 | 36.71 (8.15 to 72.80) |
| 2021–2023 | -6.87 (-22.42 to 11.79) |
| **NH Black or African American** | |
| 1999–2008 | 5.68 (4.65 to 6.72) |
| 2008–2018 | -2.56 (-3.32 to -1.80) |
| 2018–2021 | 28.59 (19.39 to 38.51) |
| 2021–2023 | 0.69 (-4.65 to 6.34) |
| **NH White** | |
| 1999–2009 | 8.23 (4.80 to 11.76) |
| 2009–2019 | 0.84 (-1.74 to 3.48) |
| 2019–2023 | 20.46 (12.32 to 29.20) |
| **Hispanic or Latino** | |
| 1999–2018 | 2.85 (2.10 to 3.61) |
| 2018–2021 | 25.17 (8.48 to 44.42) |
| 2021–2023 | -10.64 (-20.20 to 0.05) |
| **Census Region 1: Northeast** | |
| 1999–2010 | 4.33 (2.19 to 6.53) |
| 2010–2018 | -3.25 (-6.67 to 0.30) |
| 2018–2023 | 19.03 (13.15 to 25.22) |
| **Census Region 2: Midwest** | |
| 1999–2009 | 7.69 (5.09 to 10.35) |
| 2009–2019 | 0.40 (-1.79 to 2.63) |
| 2019–2023 | 20.30 (13.40 to 27.62) |
| **Census Region 3: South** | |
| 1999–2010 | 7.68 (6.96 to 8.40) |
| 2010–2018 | -1.11 (-2.08 to -0.14) |
| 2018–2021 | 25.77 (18.01 to 34.04) |
| 2021–2023 | 1.03 (-3.59 to 5.87) |
| **Census Region 4: West** | |
| 1999–2010 | 8.89 (5.13 to 12.79) |
| 2010–2018 | 0.23 (-4.46 to 5.16) |
| 2018–2023 | 15.74 (8.59 to 23.37) |
| **Metropolitan** | |
| 1999–2008 | 7.69 (5.40 to 10.03) |
| 2008–2020 | 0.90 (-0.07 to 1.88) |
| **Non-metropolitan** | |
| 1999–2011 | 8.25 (7.25 to 9.26) |
| 2011–2018 | 0.59 (-1.33 to 2.55) |
| 2018–2020 | 9.90 (-0.94 to 21.91) |
| **Young Adult** | |
| 1999–2018 | 4.45 (3.89 to 5.02) |
| 2018–2021 | 35.65 (18.28 to 55.58) |
| 2021–2023 | -6.28 (-14.88 to 3.18) |
| **Middle Aged Adult** | |
| 1999–2015 | 5.78 (5.06 to 6.50) |
| 2015–2018 | -5.63 (-16.44 to 6.57) |
| 2018–2021 | 36.56 (21.45 to 53.55) |
| 2021–2023 | -5.99 (-13.98 to 2.74) |
| **Older Adult** | |
| 1999–2009 | 7.65 (4.96 to 10.42) |
| 2009–2019 | -0.26 (-2.44 to 1.97) |
| 2019–2023 | 19.46 (12.69 to 26.64) |

**Supplementary Table 8:** Summary APCs of Sespsis and AKI-associated AAMR per 100,000 in the United States from 1999-2023.
