# Supplementary material for: Sepsis and acute kidney injury-related mortality in the U.S.: National trends and disparities (1999–2023)
Source: Medicine (Baltimore). 2026 Jun 26;105(26):e49495. doi: 10.1097/MD.0000000000049495 (PMC13313787; doi:10.1097/MD.0000000000049495)
Supplement: Supplementary file 9 [file medi-105-e49495-s009.docx]

| **Cause** | **Deaths** |
| --- | --- |
| Septicemia | 66555 |
| Nephritis, nephrotic syndrome and nephrosis | 52019 |
| Malignant neoplasms | 41500 |
| Diseases of heart | 28252 |
| Influenza and pneumonia | 24247 |
| COVID-19 | 20189 |
| Chronic liver disease and cirrhosis | 13844 |
| Diabetes mellitus | 10274 |
| Enterocolitis due to Clostridium difficile | 9209 |
| Chronic lower respiratory diseases | 8680 |
| Cerebrovascular diseases | 7406 |
| Pneumonitis due to solids and liquids | 5692 |
| Cholelithiasis and other disorders of gallbladder | 4802 |
| Accidents (unintentional injuries) | 3110 |
| Alzheimer disease | 2565 |
| Essential hypertension and hypertensive renal disease | 502 |

**Supplementary Table 9:** (UCD15) Leading Underlying Causes of Death related to Sepsis and AKI in The United States from 1999-2023
